# Evasin‐displaying lactic acid bacteria bind different chemokines and neutralize CXCL8 production in Caco‐2 cells

**DOI:** 10.1111/1751-7915.12781

**Published:** 2017-07-24

**Authors:** Katja Škrlec, Anja Pucer Janež, Boris Rogelj, Borut Štrukelj, Aleš Berlec

**Affiliations:** ^1^ Department of Biotechnology Jožef Stefan Institute Jamova 39 SI‐1000 Ljubljana Slovenia; ^2^ Graduate School of Biomedicine Faculty of Medicine University of Ljubljana SI‐1000 Ljubljana Slovenia; ^3^ Biomedical Research Institute (BRIS) Puhova 10 SI‐1000 Ljubljana Slovenia; ^4^ Faculty of Chemistry and Chemical Technology University of Ljubljana Večna pot 113 SI‐1000 Ljubljana Slovenia; ^5^ Faculty of Pharmacy University of Ljubljana Aškerčeva 7 SI‐1000 Ljubljana Slovenia

## Abstract

Chemokines are key signals in the immune system and play an important role as proinflammatory mediators in the pathology of inflammatory bowel disease and colorectal cancer, making them an important target for therapy. Recombinant lactic acid bacteria (LAB) were engineered to bind CC and CXC chemokines by displaying chemokine‐binding proteins evasin‐1, evasin‐3 and evasin‐4 on their surface. Evasin genes were cloned into lactococcal surface display vector and overexpressed in *L. lactis* NZ9000 and NZ9000*ΔhtrA* in fusion with secretion signal and surface anchor. Evasin‐displaying bacteria removed from 15% to 90% of 11 different chemokines from the solution as determined with ELISA and Luminex multiplexing assays, whereby *L. lactis* NZ9000*ΔhtrA* proved more efficient. *Lactobacillus salivarius* ATCC 11741 was coated with *L. . lactis*‐expressed evasin fusion protein, and its ability to bind chemokines was also confirmed. Evasin‐3‐displaying *L. lactis* removed 76.0% of IL‐1β‐induced CXCL8 from the supernatant of Caco‐2 epithelial cells. It also prevented secretion of CXCL8 from Caco‐2 cells in a time‐dependent manner when added before induction with IL‐1β. Evasin‐displaying LAB have the ability to bind multiple chemokines simultaneously and exert synergistic activity. This innovative treatment approach therefore has the potential for mucosal therapy of inflammatory bowel disease or colorectal cancer.

## Introduction

Chemokines are small chemoattractant cytokines acting via seven transmembrane G protein‐coupled receptors (GCPRs) that selectively induce recruitment and activation of immune cells to the site of infection. They are classified into four subfamilies, C, CC, CXC and CX3C, on the basis of the number and spacing of the conserved cysteine residues in the amino‐terminus of the protein (Zlotnik and Yoshie, 2000). Historically, their name derived from their function (e.g. macrophage inflammatory protein: MIP‐1α, β), while recently, a generic nomenclature has been applied (e.g. CCL1, CCL2). Chemokines and chemokine receptors play an important role in injury, inflammation, wound repair and cancer (Proudfoot *et al*., 2010) and are useful as diagnostic, prognostic and therapeutic targets (Castellani *et al*., 2007). Local mucosal recruitment and activation of neutrophils is a fundamental event in the pathogenesis of inflammatory bowel disease (IBD) and is mediated by interaction of chemokines with chemokine receptors on their target cells (Ajuebor and Swain, 2002; Wang *et al*., 2009; Atreya and Neurath, 2010). Expression of several chemokines, especially CXCL8, and also of the corresponding receptors, CXCR1 and CXCR2, is constantly increased during the active phase of IBD (Raab *et al*., 1993). Besides CXCL8, the levels of CXC chemokines CXCL1, CXCL5 and CXCL6 have been shown to be positively correlated with severity of inflammation in IBD patients (Ina *et al*., 1997). Additionally, recurrent inflammation and tissue destructive lesions that are accompanied by uncontrolled activation of effector immune cells in mucosa are associated with increased risk of colorectal cancer (CRC) (Triantafillidis *et al*., 2009; Sebastian *et al*., 2014). The levels of proinflammatory chemokines, such as CCL2, CCL3, CCL4 and CCL5, and pro‐angiogenic chemokines, such as CXCL1, CXCL5 and CXCL8, are also elevated in human colon tumour tissues as compared to the matched normal tissues, indicating that these chemokines and their receptors play an important role in regulating colon tumour progression, angiogenesis and metastasis (Baier *et al*., 2005). The development of chemokine‐binding proteins as potential therapeutic agents in IBD, or as preventive agents in inflammation‐associated colorectal cancer, is therefore of great interest (Triantafillidis *et al*., 2011).

Many pathogenic organisms have developed chemokine and cytokine mediators with the ability to interfere with the host chemokine network and escape host detection and defence systems (Proudfoot *et al*., 2015). Evasins belong to a family of small chemokine‐binding proteins (CKBPs). They have been identified in the salivary gland of the brown tick *Rhipicephalus sanguineus* and are probably used by the tick to inhibit the chemokine‐mediated recruitment of leucocytes to the bite site (Frauenschuh *et al*., 2007). CKBPs have also been identified in several other organisms, including viruses, worms and arthropods (Seet and McFadden, 2002; Gonzalez‐Motos *et al*., 2016). Unlike other CKBPs, evasins are smaller proteins (7–11 kDa) and display a more selective profile and a unique mechanism of chemokine binding (Deruaz *et al*., 2008; Bonvin *et al*., 2014). Evasins recognize and interact with chemokines with various degrees of selectivity and inhibit the binding of chemokines to their receptors and/or cell surface glycosaminoglycans. Evasin‐1 binds CCL3, CCL4 and CCL18 (Frauenschuh *et al*., 2007), evasin‐3 binds CXCL1 and CXCL8 (Deruaz *et al*., 2008), and evasin‐4 is able to interact with almost 20 chemokines of the CC subfamily, particularly with CCL3, CCL5, CCL18 and CCL21 (Deruaz *et al*., 2013). Evasins have inhibited cellular recruitment in different murine models of disease and demonstrated potent anti‐inflammatory properties (Deruaz *et al*., 2008; Castor *et al*., 2010; Russo *et al*., 2011; Braunersreuther *et al*., 2013; Copin *et al*., 2013; Montecucco *et al*., 2014; Bonvin *et al*., 2016).

Various lactic acid bacteria and other microorganisms have been suggested as a possible therapeutic approach for IBD treatment (Ng *et al*., 2009). Their therapeutic efficiency can be further improved by genetic modification of LAB (Kleerebezem and de Vos, 2011; Berlec *et al*., 2012; De Moreno de LeBlanc *et al*., 2015). Several strategies have been described including the production of antioxidant enzymes (Han *et al*., 2006), anti‐inflammatory cytokine IL‐10 (Schotte *et al*., 2000) or other anti‐inflammatory compounds (Vandenbroucke *et al*., 2010). A somewhat different strategy included the display of cytokine‐binding proteins on the surface of LAB (Ravnikar *et al*., 2010; Zadravec *et al*., 2015a,b; Berlec *et al*., 2016; Kosler *et al*., 2017), with the goal of preventing cytokine proinflammatory action. In the present research, novel therapeutic targets, chemokines, have been addressed in a similar fashion – chemokine‐binding proteins (CKBPs), evasins, were displayed on the surface of LAB and their ability to bind different chemokines was demonstrated. Engineered LAB constitute an innovative approach for the treatment of IBD or CRC.

## Results

### Expression and surface display of evasins on *L. lactis* NZ9000 and *L. lactis* NZ9000Δ*htrA*

**Figure 1:**
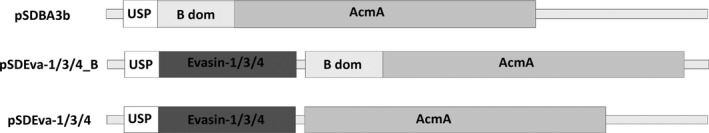
Gene constructs for lactococcal surface display. USP: gene for Usp45 signal peptide for secretion to the growth medium (84 bp). B dom: gene for reporter protein B domain of Staphylococcal protein A (174 bp). Evasin‐1/3/4: genes for chemokine‐binding evasin‐1 (327 bp), evasin‐3 (243 bp) and evasin‐4 (312 bp) respectively. AcmA: gene for C‐terminal part of AcmA protein‐containing 3 LysM repeats for surface anchoring (642 bp).

Synthetic evasin genes (Frauenschuh *et al*., 2007; Deruaz *et al*., 2008) with *L. lactis*‐optimized codon usage were cloned into the plasmid for surface display (pSDBA3b) (Ravnikar *et al*., 2010). Evasin gene constructs (Fig. 1) were expressed as fusion proteins under the control of NisA promotor. Evasin_B domain fusion proteins were composed of four functional parts, including a signal sequence for secretion to the growth medium [derived from the Usp45 (van Asseldonk *et al*., 1990)], the gene for chemokine‐binding protein (evasin‐1, evasin‐3 or evasin‐4), the gene for reporter protein B domain (IgG‐binding B domain of staphylococcal protein A) and the gene for peptidoglycan‐binding domain of AcmA (Buist *et al*., 1995) for surface attachment. Evasin fusion proteins (Fig. 1) are similar to evasin_B domain fusion proteins, lacking only the B domain reporter protein.

**Figure 2:**
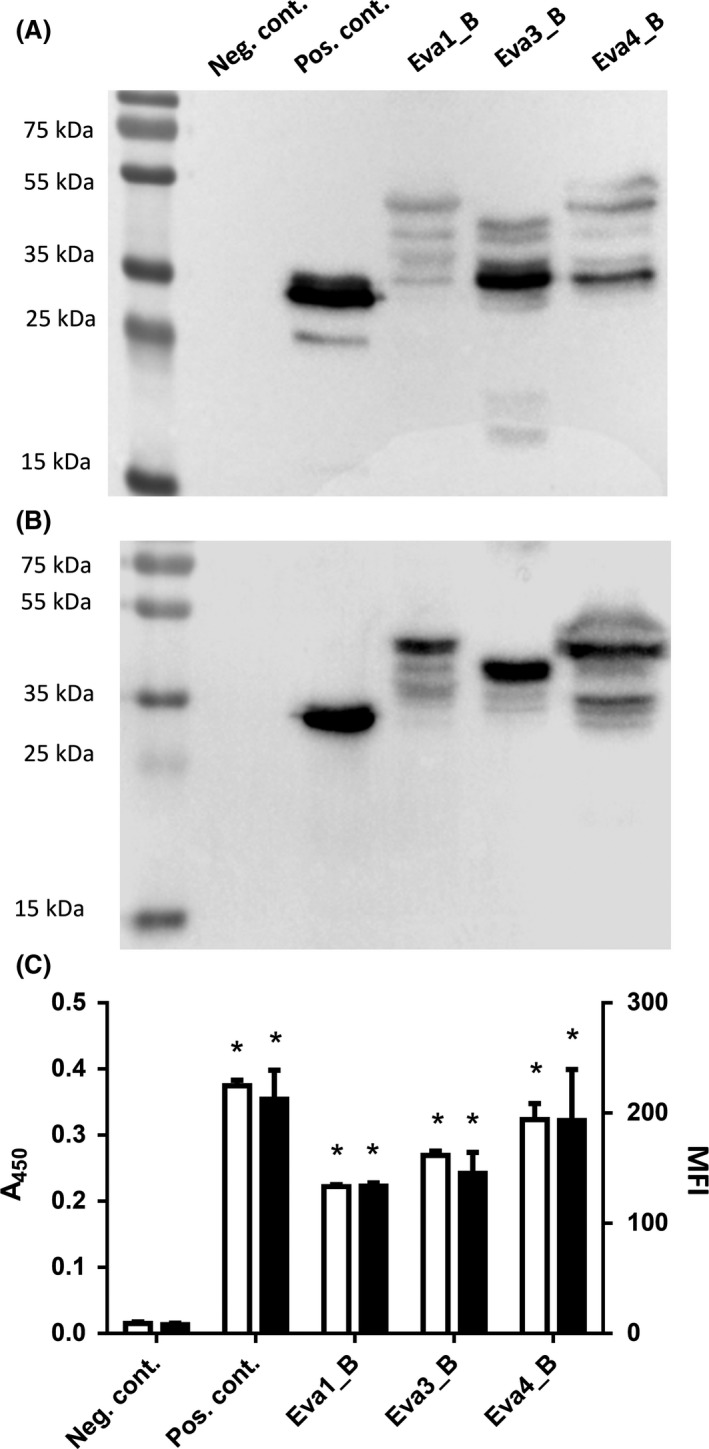
Western blot of (A) *L. lactis* NZ9000 and (B) *L. lactis* NZ9000*ΔhtrA* cells expressing evasin‐1, evasin‐3 and evasin‐4 in fusion with Usp45 secretion signal, B domain reporter protein and LysM‐containing AcmA domain. Whole‐cell ELISA (C, white bars) and flow cytometric (C, black bars) analysis of *L. lactis* NZ9000*ΔhtrA* cells expressing evasin‐1, evasin‐3 and evasin‐4 in fusion with B domain. Neg. cont.: negative control‐containing empty plasmid pNZ8148. Pos. cont.: positive control‐containing plasmid pSDBA3b (display of B domain). The results were from three independent experiments performed in triplicate and are expressed as mean ± SD. Significant difference (*P < 0.01) between evasin‐expressing cells and negative control (empty plasmid) is marked with an asterisk.

Expression of evasin_B domain fusion proteins was evaluated with specific antiprotein A antibody (recognizing B domain) using Western blot (Fig. 2). When fusion proteins were expressed in *L. lactis* NZ9000 (Fig. 2A), the bands with the highest molecular weight corresponded to the weight of the full‐length proteins (Eva‐1_B 42.2 kDa, Eva‐3_B 39.8 kDa, Eva‐4_B 43.2 kDa and B dom 32.9 kDa). The bands of lower molecular weight corresponded to degradation products. The expression of evasin_B domain fusion proteins in *L. lactis* NZ9000*ΔhtrA* (Fig. 2B) yielded bands of similar molecular weights; however, the extent of degradation was significantly lower (Fig. 2B). No expression was detected in negative control Fig 2A and B) or without nisin induction (not shown).

Surface display of evasin_B domain fusion proteins on *L. lactis* NZ9000 and *L. lactis* NZ9000*ΔhtrA* was confirmed and quantified with whole‐cell ELISA and flow cytometry (Fig. 2C), using antibodies against B domain. The extent of surface display on *L. lactis* NZ9000*ΔhtrA* is shown as it was higher than that achieved on *L. lactis* NZ9000. Statistically significant (*P *< 0.01; *t*‐test) display of evasin fusion proteins on *L. lactis* cell surface as compared to the negative control was observed with both methods. Flow cytometry of evasin_B domain fusion protein‐displaying cells showed a shift in mean fluorescence intensity (MFI) (Fig. S1) in comparison with the negative control (pNZ8148). The extent of displayed evasin_B domain fusion proteins was lower than that of displayed B domain fusion protein without evasins in the positive control. The highest extent of surface display was observed with evasin‐4_B fusion protein with both methods.

### Evaluation of chemokine binding by *L. lactis* NZ9000 and *L. lactis* NZ9000*ΔhtrA* with surface‐displayed evasins

Preliminary evaluation of chemokine‐binding ability was performed with *L. lactis* NZ9000, and the results are shown in Table 1. The chemokine selectivity of evasin‐1‐ and evasin‐4‐displaying *L. lactis* NZ9000 for CC chemokines was determined by analysing the binding of CCL3, CCL4 and CCL5 by ELISA. 2 × 10^9^ *L. lactis* NZ9000 cells with surface‐displayed evasin‐1 fusion protein bound 23% of CCL3 from the solution, but could not bind CCL5 (Table 1). 2 × 10^9^ evasin‐4‐expressing *L. lactis* NZ9000 cells removed around 40% of CCL5 from the solution, but did not bind CCL3. None of the cells bound CCL4.

**Table 1:** The percentage of CC and CXC chemokines removed from solution after incubation with 2 × 10^9^ or 1 × 10^10^ *L. lactis* NZ9000 cells that displayed on their surface evasin‐1 (pSDEva1), evasin‐4 (pSDEva4) or evasin‐3 (pSDEva3), relative to empty plasmid‐containing control cells. Chemokine concentration was determined with ELISA and multiplexing Luminex assay system MAGPIX

	*L. lactis* NZ9000		Determination methods	Number of cells
	pSDEva1	pSDEva4		
CCL3 (MIP‐1α)	23.2 ± 5.1	n.b.	ELISA	2 × 10^9^
CCL4 (MIP‐1β)	n.b	n.b.		
CCL5 (RANTES)	n.b	38.7 ± 9.6		
CCL3 (MIP‐1α)	65.8	48.5	LUMINEX	1 × 10^10^
CCL11 (Eotaxin)	n.b.	23.2		
CCL18 (PARC)	n.b.	19.5		
CCL24 (Eotaxin‐2)	n.b.	n.b.		
CCL25 (TECK)	n.b.	29.3		
	pSDEva3			
mCXCL1 (KC)	56.7 ± 7.0		ELISA	2 × 10^9^
CXCL2 (Gro‐β)	44.1 ± 1.5			
mCXCL2 (MIP‐2)	67.0 ± 6.6			
CXCL8 (IL‐8)	54.5 ± 2.1			
CXCL1 (Gro‐α)	n.b.		LUMINEX	2 × 10^9^
CXCL4 (PF4)	n.b.			
CXCL5 (ENA78)	16.7 ± 1.4			
CXCL6 (GCP‐2)	20.0 ± 4.0			
CXCL16	n.b.			

n.b., no statistically significant binding. Murine chemokines are denoted by *m*.

Apart from using ELISA, binding of chemokines CCL3 (MIP‐1α), CCL11 (Eotaxin), CCL18 (PARC), CCL24 (Eotaxin‐2) and CCL25 (TECK) was also measured simultaneously with the 5‐plex magnetic assay system MAGPIX (Table 1). 1 × 10^10^ *L. lactis* NZ9000 cells with surface‐displayed evasin‐1 bound only CCL3 from the mixture of five different chemokines. On the other hand, 1 × 10^10^ *L. lactis* NZ9000 cells with surface‐displayed evasin‐4 bound CCL3, CCL11, CCL18 and CCL25 from the mixture. No binding of CCL24 by either evasin‐1‐ or evasin‐4‐displaying *L. lactis* was observed. The presence of B domain had little effect on binding by evasin‐1‐ and evasin‐4‐displaying *L. lactis,* and the results are therefore not shown.

*L. lactis* NZ9000 cells with surface‐displayed evasin‐3 bound and removed various portions of CXC chemokines murine CXCL1 (KC), human CXCL2, murine CXCL2 (MIP‐2) and human CXCL8 (IL‐8) from the solution, as evaluated by ELISA (Table 1). The extent of chemokine removal was, in general, lower with B domain‐containing fusion protein (not shown). Human chemokines CXCL1 (Gro‐α), CXCL4 (PF4), CXCL5 (ENA78), CXCL6 (GCP‐2) and CXCL16 were evaluated simultaneously with a 5‐plex screening assay using the Luminex system. 2 × 10^9^ *L. lactis* NZ9000 cells with surface‐displayed evasin‐3 removed portions of CXCL5 and CXCL6 from the solution, while no binding of CXCL1, CXCL4 and CXCL16 was observed (Table 1).

**Figure 3:**
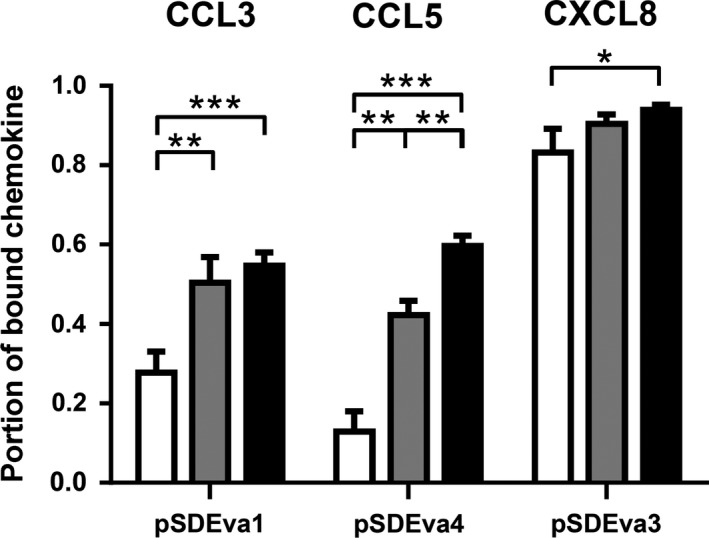
ELISA‐determined percentage of CCL3, CCL5 and CXCL8 removed after incubation with 6 × 10^8^ (white bars), 3 × 10^9^ (grey bars) or 6 × 10^9^ (black bars) cells mL^−1^ of recombinant *L. lactis* NZ9000*ΔhtrA* cells that displayed evasin‐1 (pSDEva1), evasin‐4 (pSDEva4) or evasin‐3 (pSDEva3). The results were from three independent experiments performed in triplicate and are expressed as mean ± SD. Significant differences (*P < 0.05; **P < 0.01; ***P < 0.001) are marked with asterisks.

Binding of CCL3, CCL5 and CXCL8 was studied in more detail by taking into account the influence of bacterial cell number and bacterial strain. The CCL3 and CXCL8 binding with *L. lactis* NZ9000*ΔhtrA* (Fig. 3) was 1.3‐ and 2.0‐fold better, respectively, than that achieved with *L. lactis* NZ9000 (Fig. S2), while for CCL5, the difference was less pronounced. Evasin‐1‐displaying *L. lactis* NZ9000*ΔhtrA* in concentration of 6 × 10^9^, 3 × 10^9^ and 6 × 10^8^ cells ml^−1^ removed 54.8%, 50.5% and 27.8% of CCL3 from the solution, respectively, relative to that of the control bacteria (Fig. 3). The extent of CCL5 removal by evasin‐4‐displaying *L. lactis* NZ9000*ΔhtrA* also depended on the bacterial cell number and was decreased by lowering the number of cells. Evasin‐4‐displaying *L. lactis* NZ9000*ΔhtrA* in concentration of 6 × 10^9^, 3 × 10^9^ and 6 × 10^8^ cells ml^−1^ removed 59.7%, 42.3% and 13.0% of CCL5 from the solution, respectively, relative to the control bacteria. Evasin‐3‐displaying *L. lactis* NZ9000*ΔhtrA* cells at 6 × 10^9^, 3 × 10^9^ and 6 × 10^8^ cells ml^−1^ removed 94.0%, 90.4% and 83.2%, of CXCL8, relative to the control bacteria (Fig. 3).

### Chemokine binding by *Lb. salivarius* heterologously coated with evasin fusion proteins

2 × 10^9^ *Lb. salivarius* cells were incubated with different volumes of conditioned sterile growth medium of *L. lactis* NZ9000*ΔhtrA* that contained evasin‐1, evasin‐3 and evasin‐4 fusion proteins. This resulted in heterologous non‐covalent attachment of evasin fusion proteins to the surface of *Lb. salivarius* via the peptidoglycan‐binding domain of AcmA. Binding of CCL3, CCL5 and CXCL8 was achieved by coating *Lb. salivarius* with evasin‐1, evasin‐4 and evasin‐3 fusion proteins respectively (Table 2). Decreasing the volume of the lactococcal growth medium (20, 10, 5 ml) resulted in slight, but statistically significant decrease in chemokine binding (Fig. S3).

**Table 2:** The percentage of chemokines CCL3, CCL5 and CXCL8 removed from solution after incubation with 2 × 10^9^ *Lb. salivarius* cells that were previously coated with evasin‐1 (pSDEva1), evasin‐3 (pSDEva3) and evasin‐4 (pSDEva4) fusion proteins, respectively, relative to control *Lb. salivarius* cells. Growth media of recombinant *L. lactis* NZ9000*ΔhtrA* served as a source of fusion proteins. The extent of binding was normalized relative to the control *Lb. salivarius* cells incubated with the growth medium of *L. lactis* harbouring empty plasmid pNZ8148

	*Lactobacillus salivarius* coated with growth medium of*. L. lactis* NZ9000*ΔhtrA* containing		
	pSDEva1	pSDEva3	pSDEva4
CCL3	86.6 ± 1.9	n.b.	47.0 ± 1.2
CCL5	n.b.	n.b.	49.0 ± 5.5
CXCL8	n.b.	79.7 ± 2.3	n.b.

n.b., no statistically significant binding.

### Evasin‐3‐displaying *L. lactis* and *Lb. salivarius* removed CXCL8 secreted by IL‐1β‐induced intestinal epithelial cells

Basal secretion of CXCL8 to the culture medium by untreated Caco‐2 cells was determined to be 9.5 pg ml^−1^. Treatment of Caco‐2 cells with IL‐1β resulted in increased CXCL8 production. The dose–response curve of CXCL8 secretion, following treatment of Caco‐2 cells with varying concentrations of IL‐1β (0.25–250 ng ml^−1^), showed a peak in CXCL8 production (~600 pg ml^−1^) at a concentration of 25 ng ml^−1^ IL‐1β (Fig. S4A), resulting in more than 60‐fold increase. CXCL8 secretion by Caco‐2 cells increased rapidly during first 6 h after addition of 25 ng ml^−1^ of IL‐1β (Fig. S4B).

**Figure 4:**
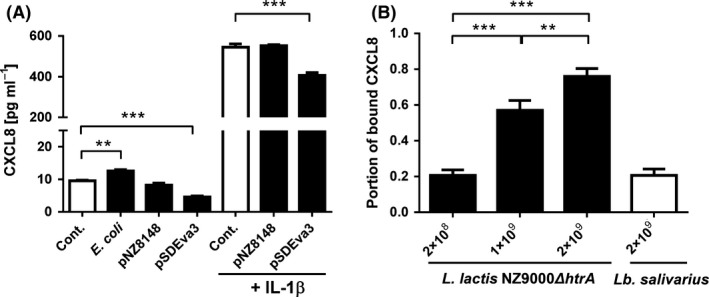
ELISA‐determined concentration (A) or portion of bound (B) CXCL8 in the supernatant of Caco‐2 cells. A. Caco‐2 cells with or without IL‐1β induction were untreated (Cont.) or were exposed to 2 × 10^9^ cells ml^−1^ of bacteria (*E. coli* DH5α, *L. lactis* NZ9000 containing empty plasmid control pNZ8148 or plasmid pSDEva3). B. Removal of CXCL8 from the supernatant of IL‐1β‐induced Caco‐2 cells by 2 × 10^8^, 1 ×10^9^ or 2 × 10^9^ cells ml^−1^ of *L. lactis* NZ9000 *ΔhtrA* (white bars) or 2 × 10^9^ cells ml^−1^ of *Lb. salivarius* cells coated with growth medium of *L. lactis* NZ9000*ΔhtrA* (black bars) displaying evasin‐3. The results were from three independent experiments performed in triplicate and are expressed as mean ± SD. Significant differences (*t*‐test) are marked with asterisks. (** < 0.01; ***P < 0.001).

The effect of evasin‐3‐displaying *L. lactis* NZ9000 on the basal secretion of CXCL8 by Caco‐2 cells (without IL‐1β addition) was compared to that of control *L. lactis* NZ9000 (containing empty plasmid pNZ8148) or wild‐type *E. coli* DH5α. 2 × 10^9^ bacterial cells ml^−1^ were used, representing a multiplicity of infection (moi) of 2000:1 (bacterial: epithelial cells). *L. lactis* NZ9000 with surface‐displayed evasin‐3 decreased CXCL8 concentration in the supernatant of Caco‐2 cells for 26.8% in comparison with empty plasmid control‐containing *L. lactis* NZ9000 (Fig. 4A), while, on the contrary, incubation with *E. coli* cells increased the CXCL8 secretion for 53.3%. Induction of CXCL8 production in Caco‐2 cells by adding 25 ng ml^−1^ of IL‐1β for 6 h, followed by 2‐h incubation with bacteria, resulted in much higher total concentrations of CXCL8. However, similar effects of bacteria were observed: control *L. lactis* NZ9000 (containing empty plasmid pNZ8148) had no effect on CXCL8 production, while evasin‐3‐displaying *L. lactis* NZ9000 caused 26.3% reduction in CXCL8 in the supernatant of Caco‐2 cells (Fig. 4A).

The bacterial removal of CXCL8 secreted from IL‐1β‐induced Caco‐2 cells was optimized by varying the number of bacterial cells and bacterial strain. Significant increase in the extent of CXCL8 removal was observed using *L. lactis* NZ9000*ΔhtrA* strain (Fig. 4B). 2 × 10^9^ evasin‐3‐expressing *L. lactis* NZ9000*ΔhtrA* reduced the concentration of CXCL8 by 76.0%, 1 × 10^9^ cells reduced the concentration of CXCL8 by 57.0%, and 2 × 10^8^ cells still reduced the concentration by 20.7% (Fig. 4B). 2 × 10^9^ *Lb. salivarius* cells coated with evasin‐3 fusion protein‐containing growth medium of *L. lactis* NZ9000*ΔhtrA* were less effective and removed only 20.7% of CXCL8 (Fig. 4B). Viability of Caco‐2 cells after incubation with bacteria was above 90% (Fig. S5A).

### Evasin‐3‐displaying *L. lactis* and *Lb. salivarius* prevented IL‐1β‐induced secretion of CXCL8 from intestinal epithelial cells

**Figure 5:**
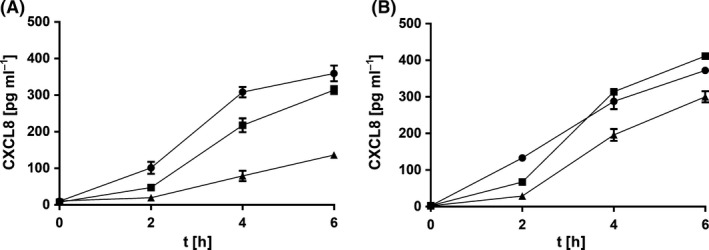
Time‐dependent secretion of CXCL8 by Caco‐2 cells after the preventive 1‐h treatment with evasin‐3‐displaying bacteria (triangles) *L. lactis* NZ9000*ΔhtrA* (A) or *Lb. salivarius* coated with evasin‐3‐fusion protein‐containing growth medium of *L. lactis* NZ9000*ΔhtrA* (B) that was followed by the addition of 25 ng ml^−1^ IL‐1β (at time 0). CXCL8 secretion by Caco‐2 cells was compared to that after incubation with control bacteria (squares; *L. lactis* NZ9000*ΔhtrA* harbouring pNZ8148 (A) or *Lb. salivarius* coated with growth medium of *L. lactis* NZ9000*ΔhtrA* cells harbouring pNZ8148 (B) or to that without bacteria (circles).

The preventive effect of evasin‐3‐displaying bacteria on CXCL8 secretion was evaluated by pre‐incubating Caco‐2 cells with bacterial cells for 1 h, prior to addition of IL‐1β. The concentration of CXCL8 was monitored over 6 h (Fig. 5). 1 × 10^9^ evasin‐3‐displaying *L. lactis* NZ9000*ΔhtrA* cells significantly impeded the time‐dependent increase in CXCL8 concentration in comparison with control cells (harbouring pNZ8148) or absence of bacteria. The final CXCL8 concentration after 6 h of monitoring was for 62% lower in the supernatant of Caco‐2 cells pre‐treated with evasin‐3‐displaying *L. lactis* NZ9000*ΔhtrA* cells than in the supernatant without bacterial cells. *Lb. salivarius* cells coated with evasin‐3 fusion protein from growth medium of *L. lactis* NZ9000*ΔhtrA* were less effective. They reduced the final CXCL8 concentration in Caco‐2 supernatant by 27% in comparison with the Caco‐2 supernatant without bacteria after 6 h of monitoring.

## Discussion

Engineered evasin‐displaying bacteria were able to bind and remove 11 different recombinant chemokines of CC and CXC subfamilies. Synergistic targeting of different chemokines and prevention of the secretion of CXCL8 by the Caco‐2 intestinal cell line suggest a significant potential of engineered bacteria in the treatment of IBD or CRC by addressing the relatively unexplored option of oral neutralization of chemokines, coupled by beneficial effects of probiotics.

Surface display of evasins on *L. lactis* was achieved using nisin‐controlled expression (Mierau and Kleerebezem, 2005) of fusion proteins consisting of chemokine‐binding proteins evasins (evasin‐1, evasin‐3 and evasin‐4) (Deruaz *et al*., 2008), C‐terminal peptidoglycan‐binding domain of AcmA (Steen *et al*., 2003) and Usp45 secretion signal (van Asseldonk *et al*., 1990; Ravnikar *et al*., 2010; Zadravec *et al*., 2015a,b, 2016). Genes for evasins were codon optimized for *L. lactis* to favour higher protein yield. A 15‐amino acid peptide linker (Gly_4_Ser)_3_ (Trinh *et al*., 2004) was included between the evasin and the peptidoglycan‐binding domain in evasin‐1 and evasin‐3 gene constructs to enable mobility and flexibility of surface‐displayed evasins. The B domain of staphylococcal protein A was included as a part of fusion proteins to serve as a reporter protein, because its effective expression and surface display in *L. lactis* has already been demonstrated (Ravnikar *et al*., 2010). Expression of evasin fusion proteins containing B domain was confirmed by Western blot. The multiple bands of lower molecular weight were a consequence of AcmA degradation (Poquet *et al*., 2000). Expression of fusion proteins in the *L. lactis* NZ9000*ΔhtrA* strain that lacks extracellular protease HtrA (Lindholm *et al*., 2004; Cortes‐Perez *et al*., 2006) resulted in significant reduction of proteolytic degradation by HtrA and higher recombinant protein yields.

Surface display of evasin fusion proteins was confirmed and its extent quantified with whole‐cell ELISA and flow cytometry by detecting B domain reporter. The extent of surface display of evasin‐B domain fusion proteins was lower than that of fusion proteins containing only B domain. This may be the consequence of lower protein yield, larger size of the fusion protein, hindered availability of B domain in the displayed fusion protein or higher proteolytic susceptibility. The level of surface display was highest with evasin‐4 fusion protein, the only fusion protein in which (Gly_4_Ser)_3_ linker was not included due to cloning issues. Although surprising, this is in agreement with our previous report in which the linker did not contribute to the extent of surface display (Zadravec *et al*., 2014). The lower level of fusion protein degradation in NZ9000*ΔhtrA* resulted in the increased extent of surface display. Although this is in accordance with expectations, it was not observed in our previous study in which only B domain was displayed (Ravnikar *et al*., 2010), possibly pointing to the susceptibility of evasins or linker to degradation. Taken together, these results demonstrate that *L. lactis* NZ9000*ΔhtrA* strain stabilizes surface‐associated proteins and is more appropriate as an expression host for evasin fusion proteins.

The functionality of all three evasins on the bacterial surface was confirmed indirectly, via the greater ability of evasin‐displaying bacterial cells to remove chemokines from the solution than that of control cells. The functional expression is noteworthy because of the presence of intramolecular disulfide bonds which are less common in proteins of Gram‐positive LAB (Davey *et al*., 2016). Among recombinant proteins, disulfide bond‐containing bacteriocins have been produced in *L. lactis*, and evasins may share the same mechanisms of disulfide bond formation (Back *et al*., 2016; Davey *et al*., 2016).

Using different evasins‐displaying bacteria, the binding of 11 different chemokines: five human CC chemokines (CCL3, CCL5, CCL11, CCL18 and CCL25), four human CXC chemokines (CXCL2, CXCL8, CXCL5 and CXCL6) and two murine CXC chemokines (CXCL1 and CXCL2) has been demonstrated. The binding specificity of evasins was in accordance with that published. The extent of chemokine removal varied from app. 15% (CXCL5) to more than 90% (CXCL8) and was largely dependent on bacterial concentration. As seen with the expression and surface display, the binding of chemokines was improved when fusion proteins were expressed in *L. lactis* NZ9000*ΔhtrA*. The presence of B domain in fusion proteins had minor, usually detrimental, effect on binding, although positive effects were also observed, pointing to the importance of steric positioning of evasins for binding of different chemokines.

CC chemokines were removed by evasin‐1‐ and evasin‐4‐displaying bacteria. Evasin‐1‐displaying bacteria were capable of binding CCL3, but not the closely related CCL5, although binding of CCL5 by evasin‐1 was previously reported (Frauenschuh *et al*., 2007). This may be the consequence of insufficient concentration of cells, or incorporation of evasin‐1 into the fusion protein via its C‐terminus, as it was shown that C‐terminus was involved in chemokine binding (in contrast to evasin‐4 that binds chemokines via its N‐terminus) (Deruaz *et al*., 2013). Evasin‐4 has a broader selectivity pattern than evasin‐1 and evasin‐3 and was previously shown to bind almost 20 chemokines of the CC subfamily, among them CCL5 with the highest affinity (Deruaz *et al*., 2013). Removal of CCL5 by evasin‐4‐displaying *L. lactis* cells was indeed very effective (42.3%). Apart from CCL5, evasin‐4‐displaying cells also bound CCL3, CCL11, CCL18 and CCL25 in a multiplex Luminex assay.

CXC chemokines (human CXCL2, CXCL5, CXCL6, CXCL8 and mouse homologues CXCL1 and CXCL2) were bound by evasin‐3‐displaying *L. lactis* cells. Binding of human CXCL1 was not detected, possibly due to the multiplex Luminex assay format and the competition by five different chemokines. This could also be the reason for the generally lower percentage of binding detected with Luminex. Detailed characterization of binding with evasin‐3‐displaying *L. lactis* cells was performed with CXCL8. As observed with evasin‐1‐ or evasin‐4‐displaying cells, binding of CXCL8 was more effective with higher number of evasin‐3‐displaying bacterial cells.

*Lactobacillus salivarius* ATCC 11741 has been established earlier as a suitable host for heterologous surface display of proteins containing the peptidoglycan‐binding domain of AcmA (Zadravec *et al*., 2015a,b; Kosler *et al*., 2017). It was shown that not all AcmA‐containing fusion protein, produced and secreted by recombinant *L. lactis,* binds to the producer cells. The fusion protein‐containing growth medium can therefore be used for heterologous coating of non‐recombinant bacterial cells (Bosma *et al*., 2006), representing a non‐GMO alternative (Zadravec *et al*., 2015a,b) or a surface display option for hard to transform bacteria. *Lb. salivarius* was coated with evasin fusion proteins produced by *L. lactis,* and its ability to bind chemokines was confirmed.

Inflammation of the intestinal epithelial cells is usually driven by inflammatory cytokines from the immune cells of the lamina propria. This can be recreated in the Caco‐2 epithelial cell model by stimulation with IL‐1β which triggers strong secretion of CXCL8 (Luerce *et al*., 2014) that can accumulate in the growth medium for several hours. Evasin‐3‐displaying *L. lactis* significantly reduced CXCL8 concentration (by up to 76.0%) in Caco‐2 cell supernatant in comparison with untransformed *L. lactis* or *E. coli*. Evasin‐3‐coated *Lb. salivarius* was less effective in binding Caco‐2‐produced CXCL8 than evasin‐3‐displaying *L. lactis,* despite their similar binding abilities as observed with ELISA (Tables 1 and 2). This might be due to the intrinsic slightly proinflammatory phenotype of *Lb. salivarius* that was shown to be able to induce TNFα production in THP‐1 cells (Diaz *et al*., 2013). However, *Lb. salivarius* coated with evasin‐3 fusion protein‐containing NZ9000*ΔhtrA* growth medium could still remove around 20% of CXCL8 from the Caco‐2 supernatant.

Preventive addition of evasin‐3‐displaying *L. lactis* to Caco‐2 cells for 1 h before exposure to IL‐1β was shown to reduce CXCL8 secretion in a time‐dependent manner. A less intense, but still significant decrease in CXCL8 was observed with control *L. lactis,* in line with the intrinsic anti‐inflammatory activity of *L. lactis* (Berlec *et al*., 2016). The decrease achieved with evasin‐3‐displaying *Lb. salivarius* was again less pronounced than that achieved with evasin‐3‐displaying *L. lactis*.

The preventive addition of chemokine‐binding bacteria to the epithelial cell model resembles the intended administration of bacteria in intestinal inflammations and suggests a significant potential for mucosal therapy of inflammation occurring in IBD or colorectal cancer. This potential is strengthened by the intrinsic evasin affinity for different chemokines. The chemokine system is promiscuous, with individual chemokines binding to several receptors and individual receptors recognizing multiple chemokines (Allegretti *et al*., 2012; Steen *et al*., 2014). Targeting a single chemokine or single receptor would therefore not be sufficient to exert a significant effect *in vivo* (Koelink *et al*., 2012). However, targeting multiple chemokines with three different evasins could avoid redundancy and enable synergy (Bonvin *et al*., 2016). The ability of evasin‐displaying bacteria to bind multiple cytokines simultaneously has been confirmed in this study using multiplex assays.

To conclude, we present for the first time the expression of biologically active evasins and their display on the surface of LAB. We have shown that evasin‐displaying LAB are able to bind at least 11 different chemokines, some of them at the same time. Apart from binding the recombinant chemokines, the evasin‐displaying bacteria also prevented CXCL8 secretion in the epithelial cell model. The results obtained thus warrant expanding the studies on chemokine‐binding bacteria to animal models of intestinal inflammation with the aim of confirming their usefulness in the treatment of IBD and CRC.

## Experimental procedures

### Bacterial strains, media and growth conditions

The bacterial strains used in this study are shown in Table S1. *Lactococcus lactis* NZ9000 (de Ruyter *et al*., 1996; Kuipers *et al*., 1998; Mierau and Kleerebezem, 2005) and *Lactococcus lactis* NZ9000Δ*htrA* (Lindholm *et al*., 2004) were grown at 30 °C in M17 medium (Sigma) supplemented with 0.5% glucose (GM‐17) without agitation or in the same medium solidified with 1.5% agar. To maintain selection pressure on transformation, 10 μg ml^−1^ chloramphenicol or erythromycin, or both, was added to the growth medium of *L. lactis* NZ9000 and *L. lactis* NZ9000*ΔhtrA*. *Lactobacillus salivarius* ATCC 11741 was grown in De Man, Rogosa and Sharpe (MRS) medium (Merck) at 37 °C without aeration. *E. coli* strain DH5α was grown at 37 °C with agitation in lysogeny broth (LB) medium supplemented with 100 μg ml^−1^ ampicillin.

### DNA manipulation and plasmid construction

Detailed cloning procedures are described in Supporting information. Primers (IDT) and plasmids are listed in Table S1. Scheme of evasin constructs pSDEva1, pSDEva3 and pSDEva4 is shown in Fig. 1.

### Expression of evasin fusion proteins in *L. lactis*

Overnight cultures of *L. lactis* NZ9000 and *L. lactis* NZ9000*ΔhtrA* harbouring plasmids pNZ8148, pSDBA3b, pSDEva1, pSDEva1_B, pSDEva3, pSDEva3_B, pSDEva4 and pSDEva4_B were diluted (1:100) in 10 ml (or 100 ml) of fresh GM‐17 medium and grown to an optical density (A_600_) of 0.50–0.80. Fusion protein expression was induced with 25 ng ml^−1^ nisin (Fluka) (de Ruyter *et al*., 1996; Kuipers *et al*., 1998; Mierau and Kleerebezem, 2005). After 3 h of incubation, 1 ml of culture was stored at 4 °C for flow cytometric analysis, and the remaining cell culture was centrifuged at 5000 *g* for 10 min. The cell pellet was resuspended in 400 μl of phosphate‐buffered saline (PBS, pH 7.4) and stored at −20 °C for SDS‐PAGE analysis, or resuspended in different volumes of PBS for whole‐cell ELISA and assay of the chemokine‐binding ability. The supernatant was decanted, filtered with a 0.22 μm pore size filter (Minisart; Millipore, Darmstadt, Germany), aliquoted and stored at −20 °C for testing the heterologous coating of non‐recombinant bacteria with evasin fusion proteins.

### Heterologous coating of *Lactobacillus salivarius* with evasin fusion proteins

The coating of *Lb. salivarius* was performed as described previously (Zadravec *et al*., 2015a,b; Kosler *et al*., 2017). *Lb. salivarius* overnight culture containing 2 × 10^9^ cells ml^−1^ was centrifuged (5000 *g*, 5 min, 4 °C) and resuspended in different volumes (10 ml in Caco‐2 cell experiments) of the conditioned growth medium of *L. lactis* NZ9000 or *L. lactis* NZ9000*ΔhtrA* that contained evasin fusion proteins. Bacterial suspension was shaken gently for 2 h at room temperature (RT). After centrifugation (5000 *g*, 5 min, 4 °C), the cells were resuspended in PBS for assaying the chemokine‐binding ability with ELISA as described below. The growth medium of empty plasmid (pNZ8148)‐containing *L. lactis* was used as a control and was incubated with *Lb. salivarius* in the same manner.

### SDS‐PAGE and Western blot

Detailed procedure is described in Supporting information.

### Flow cytometry

Details of flow cytometry are described in Supporting information.

### Whole‐cell enzyme‐linked immunosorbent assay (ELISA)

The whole‐cell enzyme‐linked immunosorbent assay (ELISA) was performed as described previously (Lindholm *et al*., 2004; Zadravec *et al*., 2015a,b). Absorbances were read at 450 nm using an Infinite M1000 (Tecan, Salzburg, Austria). Detailed procedure is described in Supporting information.

### Chemokine binding by evasin‐displaying lactic acid bacteria

Different volumes of *L. lactis* NZ9000 and *L. lactis* NZ9000*ΔhtrA* expressing evasin‐1, evasin‐3 or evasin‐4 and *Lactobacillus salivarius* ATCC 11741 coated with evasin‐1, evasin‐3 and evasin‐4 fusion proteins were centrifuged (5000 *g*, 5 min, 4 °C), washed twice with 500 μl PBS and finally resuspended in 200 μl of PBS containing various concentrations of chemokine standards (from ELISA kits, see below) and incubated 2 h at room temperature (RT) with gentle shaking. Cells were then removed by centrifugation (5000 *g*, 10 min, 4 °C) and 100 μl of the supernatant collected to examine the content of chemokines using ELISA kits (Mabtech, Nacka Strand, Sweden; R&D Systems, Minneapolis, MN, USA; and PeproTech, London, UK). Alternatively, 50 μl of the supernatant was used to examine the content of chemokines in multiplexing system (Luminex 100 or MAGPIX, 's‐Hertogenbosch, The Netherlands). The chemokine‐binding ability of evasin‐displaying bacteria was presented as the portion of cytokine removed from the solution by evasin‐displaying bacteria, in comparison with control bacteria, harbouring pNZ8148 plasmid.

### ELISA for chemokine concentration determination

CXCL8 and CCL4 were measured using a Human IL‐8 (CXCL8) and Human MIP‐1β (CCL4) ELISA development kit (Mabtech) following the manufacturer's instructions. CCL3 (MIP‐1α) and CCL5 (RANTES) were measured using DuoSet ELISA (R&D Systems). CXCL2 (Gro‐β), CXCL16, murine CXCL1 (KC) and murine CXCL2 (MIP‐2) were measured using mini ELISA development kits (PeproTech). Standard curves for chemokines and detailed procedure of ELISA are described in Supporting information.

### Luminex multiplexing system assays for chemokine concentration determination

Human chemokines CXCL1 (Gro‐α), CXCL4 (PF4), CXCL5 (ENA78), CXCL6 (GCP‐2), CXCL8 and CXCL16 levels were measured using Luminex screening human assay kit, the xMAP Luminex fluorescent bead‐based technology (R&D Systems) according to manufacturer's instructions, and fluorescence signal was read on a Luminex 100 System (Luminex). The trimmed median value was used to derive the standard curve and calculate sample concentration. Data from three independent experiments were considered. Human chemokines CCL3 (MIP‐1α), CCL11 (Eotaxin), CCL18 (PARC), CCL24 (Eotaxin‐2) and CCL25 (TECK) were measured using a 5‐plex magnetic Luminex screening assay (R&D Systems). The samples were assayed in MAGPIX system, and data were analysed with xPONENT software (Luminex). Standard curves for chemokines are described in Supporting information.

### Caco‐2 cell culturing and incubation with bacteria

Caco‐2 cells (ATCC HTB‐37), a human colon adenocarcinoma cell line, were cultured in Dulbecco's modified Eagle's medium (DMEM, high glucose) with GlutaMAX (Gibco, Life Technologies, Paisley, UK) supplemented with 10% (v/v) fetal bovine serum (FBS) (Gibco, Life Technologies), 25 mM HEPES (Sigma‐Aldrich, St. Louis, MO, USA), 100 U m^−1^ penicillin, 100 μg ml^−1^ streptomycin (Gibco, Life Technologies) and 1% Eagle's minimum essential medium (MEM) non‐essential amino acid solution (Sigma‐Aldrich) in a humidified atmosphere containing 5% CO_2_ at 37 °C.

Caco‐2 cells were seeded at 1 × 10^5^ cells per well in 24‐well plates and incubated at 37 °C with 5% CO_2_ for 24 h before treatment, as reported (Luerce *et al*., 2014). Secretion of CXCL8 was induced by the addition of different concentrations (250, 25, 2.5, 0.25, 0.025, 0.0025, 0.00025 ng ml^−1^) of recombinant human IL‐1β (Cell Genix, Freiburg, Germany). To examine the production of CXCL8, Caco‐2 cells were incubated with 25 ng ml^−1^ of IL‐1β for different time periods (0–48 h). 25 ng ml^−1^ of IL‐1β for 6 h was used when incubating with bacteria. Cell cultures were centrifuged (10 min 200 *g* at 4 °C and 10 min 16 000 *g* at 4 °C), and the supernatant was collected and stored at −80 °C until analysis. CXCL8 levels were measured using a Human IL‐8 (CXCL8) ELISA development kit (Mabtech) as described above.

Cultures of *E. coli* DH5α, *L. lactis* NZ9000 or *L. lactis* NZ9000*ΔhtrA*, harbouring plasmids pNZ8148 (control) and pSDEva3, as well as evasin‐3‐coated *Lb. salivarius,* were centrifuged, washed two times with PBS and finally resuspended in DMEM. In CXCL8 removal experiments, bacterial cells were added to the Caco‐2 cell culture (preincubated with IL‐1β for 6 h) at a final concentration of 2 × 10^8^, 1 × 10^9^ or 2 × 10^9^ cells ml^−1^. CXCL8 secretion was measured after 2 h of co‐incubation. To prevent CXCL8 secretion, we incubated bacterial cells with Caco‐2 cells for 1 h before inducing with IL‐1β for another 6 h. Untreated Caco‐2 cells secreting baseline levels of CXCL8 were used as a control. Data from three independent experiments were considered. Viability of Caco‐2 cells after incubation with bacteria was tested with Trypan blue exclusion staining.

## Statistical analyses

All data are presented as means ± standard deviation (SD). Statistical analyses were performed using GraphPad Prism 6.01 software (San Diego, CA, USA). Student's *t*‐test was used to compare the differences. Significant differences (*t*‐test; *P < 0.05; **P < 0.01; ***P < 0.001) from the control are marked with an asterisk. The portion of removal of chemokine by bacteria was calculated only when the difference in chemokine concentration between evasin‐displaying and control bacteria was significant (*t*‐test; *P* < 0.05).

## Conflict of interest

None declared.

## Supporting information


**Table S1.** Strains, plasmids and primers used in the study.
**Fig. S1.** Shift in fluorescence intensity.
**Fig. S2.** ELISA‐determined percentage of CCL3, CCL5 and CXCL8 removed after incubation with 6 × 10^8^ (white bars), 3 × 10^9^ (gray bars), or 6 × 10^9^ (black bars) cells/mL of recombinant *L. lactis* NZ9000 cells that displayed evasin‐1 (pSDEva1), evasin‐4 (pSDEva4), or evasin‐3 (pSDEva3).
**Fig. S3.** The portion of CCL3 removed from the solution after incubation with 2 x 10^9^ *Lb. salivarius* cells coated with evasin‐1 (white bars) or evasin‐4 (black bars) fusion proteins.
**Fig. S4.** ELISA‐determined CXCL8 secretion by Caco‐2 cells exposed to IL‐1β.
**Fig. S5.** Percentage of viable Caco‐2 cells determined with trypan blue exclusion staining after 2 h of incubation with different number of *L. lactis* NZ9000*ΔhtrA* cells (2 × 10^8^, 1 × 10^9^ and 2 × 10^9^; A); or after 7 h of incubation of Caco‐2 cells with bacterial cells (*L. lactis* NZ9000*ΔhtrA* and *Lb. salivarius* coated with conditioned medium of *L. lactis* NZ9000*ΔhtrA*; B).
**Data S1.** Supplemental methods.

## References

[mbt212781-bib-0001] Ajuebor, M.N. , and Swain, M.G. (2002) Role of chemokines and chemokine receptors in the gastrointestinal tract. Immunology 105: 137–143. 11872088 10.1046/j.1365-2567.2002.01309.x PMC1782653

[mbt212781-bib-0002] Allegretti, M. , Cesta, M.C. , Garin, A. , and Proudfoot, A.E. (2012) Current status of chemokine receptor inhibitors in development. Immunol Lett 145: 68–78. 22698186 10.1016/j.imlet.2012.04.003

[mbt212781-bib-0003] van Asseldonk, M. , Rutten, G. , Oteman, M. , Siezen, R.J. , de Vos, W.M. , and Simons, G. (1990) Cloning of *usp45*, a gene encoding a secreted protein from *Lactococcus lactis* subsp. *lactis* MG1363. Gene 95: 155–160. 2123812 10.1016/0378-1119(90)90428-t

[mbt212781-bib-0004] Atreya, R. , and Neurath, M.F. (2010) Chemokines in inflammatory bowel diseases. Dig Dis 28: 386–394. 20926862 10.1159/000320392

[mbt212781-bib-0005] Back, A. , Borges, F. , Mangavel, C. , Paris, C. , Rondags, E. , Kapel, R. , *et al* (2016) Recombinant pediocin in *Lactococcus lactis*: increased production by propeptide fusion and improved potency by co‐production with PedC. Microb Biotechnol 9: 466–477. 26147827 10.1111/1751-7915.12285 PMC4919988

[mbt212781-bib-0006] Baier, P.K. , Eggstein, S. , Wolff‐Vorbeck, G. , Baumgartner, U. , and Hopt, U.T. (2005) Chemokines in human colorectal carcinoma. Anticancer Res 25: 3581–3584. 16101183

[mbt212781-bib-0007] Berlec, A. , Ravnikar, M. , and Strukelj, B. (2012) Lactic acid bacteria as oral delivery systems for biomolecules. Pharmazie 67: 891–898. 23210237

[mbt212781-bib-0008] Berlec, A. , Perse, M. , Ravnikar, M. , Lunder, M. , Erman, A. , Cerar, A. , and Strukelj, B. (2016) Dextran sulphate sodium colitis in C57BL/6J mice is alleviated by *Lactococcus lactis* and worsened by the neutralization of Tumor necrosis Factor alpha. Int Immunopharmacol 43: 219–226. 28039805 10.1016/j.intimp.2016.12.027

[mbt212781-bib-0009] Bonvin, P. , Dunn, S.M. , Rousseau, F. , Dyer, D.P. , Shaw, J. , Power, C.A. , *et al* (2014) Identification of the pharmacophore of the CC chemokine‐binding proteins Evasin‐1 and ‐4 using phage display. J Biol Chem 289: 31846–31855. 25266725 10.1074/jbc.M114.599233 PMC4231662

[mbt212781-bib-0010] Bonvin, P. , Power, C.A. , and Proudfoot, A.E. (2016) Evasins: therapeutic potential of a new family of chemokine‐binding proteins from ticks. Front Immunol 7: 208. 27375615 10.3389/fimmu.2016.00208 PMC4894869

[mbt212781-bib-0011] Bosma, T. , Kanninga, R. , Neef, J. , Audouy, S.A. , van Roosmalen, M.L. , Steen, A. , *et al* (2006) Novel surface display system for proteins on non‐genetically modified gram‐positive bacteria. Appl Environ Microbiol 72: 880–889. 16391130 10.1128/AEM.72.1.880-889.2006 PMC1352190

[mbt212781-bib-0012] Braunersreuther, V. , Montecucco, F. , Pelli, G. , Galan, K. , Proudfoot, A.E. , Belin, A. , *et al* (2013) Treatment with the CC chemokine‐binding protein Evasin‐4 improves post‐infarction myocardial injury and survival in mice. Thromb Haemost 110: 807–825. 23925450 10.1160/TH13-04-0297

[mbt212781-bib-0013] Buist, G. , Kok, J. , Leenhouts, K.J. , Dabrowska, M. , Venema, G. , and Haandrikman, A.J. (1995) Molecular cloning and nucleotide sequence of the gene encoding the major peptidoglycan hydrolase of *Lactococcus lactis*, a muramidase needed for cell separation. J Bacteriol 177: 1554–1563. 7883712 10.1128/jb.177.6.1554-1563.1995 PMC176772

[mbt212781-bib-0014] Castellani, M.L. , Bhattacharya, K. , Tagen, M. , Kempuraj, D. , Perrella, A. , De Lutiis, M. , *et al* (2007) Anti‐chemokine therapy for inflammatory diseases. Int J Immunopathol Pharmacol 20: 447–453. 17880758 10.1177/039463200702000303

[mbt212781-bib-0015] Castor, M.G. , Rezende, B. , Resende, C.B. , Alessandri, A.L. , Fagundes, C.T. , Sousa, L.P. , *et al* (2010) The CCL3/macrophage inflammatory protein‐1 alpha‐binding protein evasin‐1 protects from graft‐versus‐host disease but does not modify graft‐versus‐leukemia in mice. J Immunol 184: 2646–2654. 20100934 10.4049/jimmunol.0902614

[mbt212781-bib-0016] Copin, J.C. , da Silva, R.F. , Fraga‐Silva, R.A. , Capettini, L. , Quintao, S. , Lenglet, S. , *et al* (2013) Treatment with Evasin‐3 reduces atherosclerotic vulnerability for ischemic stroke, but not brain injury in mice. J Cereb Blood Flow Metab 33: 490–498. 23250107 10.1038/jcbfm.2012.198 PMC3618389

[mbt212781-bib-0017] Cortes‐Perez, N.G. , Poquet, I. , Oliveira, M. , Gratadoux, J.J. , Madsen, S.M. , Miyoshi, A. , *et al* (2006) Construction and characterization of a *Lactococcus lactis* strain deficient in intracellular ClpP and extracellular HtrA proteases. Microbiol 152: 2611–2618. 10.1099/mic.0.28698-0 16946256

[mbt212781-bib-0018] Davey, L. , Halperin, S.A. , and Lee, S.F. (2016) Thiol‐disulfide exchange in Gram‐positive *Firmicutes* . Trends Microbiol 24: 902–915. 27426970 10.1016/j.tim.2016.06.010

[mbt212781-bib-0019] de Moreno de LeBlanc, A. , Del Carmen, S. , Chatel, J.M. , Miyoshi, A. , Azevedo, V. , Langella, P. , *et al* (2015) Current review of genetically modified lactic acid bacteria for the prevention and treatment of colitis using murine models. Gastroenterol Res Pract 2015: 146972. 26064086 10.1155/2015/146972 PMC4434185

[mbt212781-bib-0020] Deruaz, M. , Frauenschuh, A. , Alessandri, A.L. , Dias, J.M. , Coelho, F.M. , Russo, R.C. , *et al* (2008) Ticks produce highly selective chemokine binding proteins with antiinflammatory activity. J Exp Med 205: 2019–2031. 18678732 10.1084/jem.20072689 PMC2526197

[mbt212781-bib-0021] Deruaz, M. , Bonvin, P. , Severin, I.C. , Johnson, Z. , Krohn, S. , Power, C.A. , and Proudfoot, A.E. (2013) Evasin‐4, a tick‐derived chemokine‐binding protein with broad selectivity can be modified for use in preclinical disease models. FEBS J 280: 4876–4887. 23910450 10.1111/febs.12463 PMC4240464

[mbt212781-bib-0022] Diaz, M.A. , Bik, E.M. , Carlin, K.P. , Venn‐Watson, S.K. , Jensen, E.D. , Jones, S.E. , *et al* (2013) Identification of *Lactobacillus* strains with probiotic features from the bottlenose dolphin (*Tursiops truncatus*). J Appl Microbiol 115: 1037–1051. 23855505 10.1111/jam.12305 PMC4063339

[mbt212781-bib-0023] Frauenschuh, A. , Power, C.A. , Deruaz, M. , Ferreira, B.R. , Silva, J.S. , Teixeira, M.M. , *et al* (2007) Molecular cloning and characterization of a highly selective chemokine‐binding protein from the tick *Rhipicephalus sanguineus* . J Biol Chem 282: 27250–27258. 17640866 10.1074/jbc.M704706200

[mbt212781-bib-0024] Gonzalez‐Motos, V. , Kropp, K.A. , and Viejo‐Borbolla, A. (2016) Chemokine binding proteins: an immunomodulatory strategy going viral. Cytokine Growth Factor Rev 30: 71–80. 26987612 10.1016/j.cytogfr.2016.02.007

[mbt212781-bib-0025] Han, W. , Mercenier, A. , Ait‐Belgnaoui, A. , Pavan, S. , Lamine, F. , Van Swam, I.I. , *et al* (2006) Improvement of an experimental colitis in rats by lactic acid bacteria producing superoxide dismutase. Inflamm Bowel Dis 12: 1044–1052. 17075345 10.1097/01.mib.0000235101.09231.9e

[mbt212781-bib-0026] Ina, K. , Kusugami, K. , Yamaguchi, T. , Imada, A. , Hosokawa, T. , Ohsuga, M. , *et al* (1997) Mucosal interleukin‐8 is involved in neutrophil migration and binding to extracellular matrix in inflammatory bowel disease. Am J Gastroenterol 92: 1342–1346. 9260803

[mbt212781-bib-0027] Kleerebezem, M. , and de Vos, W.M. (2011) Lactic acid bacteria: life after genomics. Microb Biotechnol 4: 318–322. 21518298 10.1111/j.1751-7915.2011.00262.x PMC3818990

[mbt212781-bib-0028] Koelink, P.J. , Overbeek, S.A. , Braber, S. , de Kruijf, P. , Folkerts, G. , Smit, M.J. , and Kraneveld, A.D. (2012) Targeting chemokine receptors in chronic inflammatory diseases: an extensive review. Pharmacol Ther 133: 1–18. 21839114 10.1016/j.pharmthera.2011.06.008

[mbt212781-bib-0029] Kosler, S. , Strukelj, B. , and Berlec, A. (2017) Lactic acid bacteria with concomitant IL‐17, IL‐23 and TNFalpha‐ binding ability for the treatment of inflammatory bowel disease. Curr Pharm Biotechnol 18: 318–326. 28190384 10.2174/1389201018666170210152218

[mbt212781-bib-0030] Kuipers, O.P. , de Ruyter, P.G.G.A. , Kleerebezem, M. , and de Vos, W.M. (1998) Quorum sensing‐controlled gene expression in lactic acid bacteria. J Biotechnol 64: 15–21.

[mbt212781-bib-0031] Lindholm, A. , Smeds, A. , and Palva, A. (2004) Receptor binding domain of *Escherichia coli* F18 fimbrial adhesin FedF can be both efficiently secreted and surface displayed in a functional form in *Lactococcus lactis* . Appl Environ Microbiol 70: 2061–2071. 15066797 10.1128/AEM.70.4.2061-2071.2004 PMC383157

[mbt212781-bib-0032] Luerce, T.D. , Gomes‐Santos, A.C. , Rocha, C.S. , Moreira, T.G. , Cruz, D.N. , Lemos, L. , *et al* (2014) Anti‐inflammatory effects of *Lactococcus lactis* NCDO 2118 during the remission period of chemically induced colitis. Gut Pathog 6: 33. 25110521 10.1186/1757-4749-6-33 PMC4126083

[mbt212781-bib-0033] Mierau, I. , and Kleerebezem, M. (2005) 10 years of the nisin‐controlled gene expression system (NICE) in *Lactococcus lactis* . Appl Microbiol Biotechnol 68: 705–717. 16088349 10.1007/s00253-005-0107-6

[mbt212781-bib-0034] Montecucco, F. , Mach, F. , Lenglet, S. , Vonlaufen, A. , Gomes Quindere, A.L. , Pelli, G. , *et al* (2014) Treatment with Evasin‐3 abrogates neutrophil‐mediated inflammation in mouse acute pancreatitis. Eur J Clin Invest 44: 940–950. 25132144 10.1111/eci.12327

[mbt212781-bib-0035] Ng, S.C. , Hart, A.L. , Kamm, M.A. , Stagg, A.J. , and Knight, S.C. (2009) Mechanisms of action of probiotics: recent advances. Inflamm Bowel Dis 15: 300–310. 18626975 10.1002/ibd.20602

[mbt212781-bib-0036] Poquet, I. , Saint, V. , Seznec, E. , Simoes, N. , Bolotin, A. , and Gruss, A. (2000) HtrA is the unique surface housekeeping protease in *Lactococcus lactis* and is required for natural protein processing. Mol Microbiol 35: 1042–1051. 10712686 10.1046/j.1365-2958.2000.01757.x

[mbt212781-bib-0037] Proudfoot, A.E. , Power, C.A. , and Schwarz, M.K. (2010) Anti‐chemokine small molecule drugs: a promising future? Expert Opin Investig Drugs 19: 345–355. 10.1517/13543780903535867 20113217

[mbt212781-bib-0038] Proudfoot, A.E. , Bonvin, P. , and Power, C.A. (2015) Targeting chemokines: pathogens can, why can't we? Cytokine 74: 259–267. 25753743 10.1016/j.cyto.2015.02.011

[mbt212781-bib-0039] Raab, Y. , Gerdin, B. , Ahlstedt, S. , and Hallgren, R. (1993) Neutrophil mucosal involvement is accompanied by enhanced local production of interleukin‐8 in ulcerative colitis. Gut 34: 1203–1206. 8406154 10.1136/gut.34.9.1203 PMC1375454

[mbt212781-bib-0040] Ravnikar, M. , Strukelj, B. , Obermajer, N. , Lunder, M. , and Berlec, A. (2010) Engineered lactic acid bacterium *Lactococcus lactis* capable of binding antibodies and tumor necrosis factor alpha. Appl Environ Microbiol 76: 6928–6932. 20802083 10.1128/AEM.00190-10 PMC2953040

[mbt212781-bib-0041] Russo, R.C. , Alessandri, A.L. , Garcia, C.C. , Cordeiro, B.F. , Pinho, V. , Cassali, G.D. , *et al* (2011) Therapeutic effects of evasin‐1, a chemokine binding protein, in bleomycin‐induced pulmonary fibrosis. Am J Respir Cell Mol Biol 45: 72–80. 20833968 10.1165/rcmb.2009-0406OC

[mbt212781-bib-0042] de Ruyter, P.G. , Kuipers, O.P. , and de Vos, W.M. (1996) Controlled gene expression systems for *Lactococcus lactis* with the food‐grade inducer nisin. Appl Environ Microbiol 62: 3662–3667. 8837421 10.1128/aem.62.10.3662-3667.1996 PMC168174

[mbt212781-bib-0043] Schotte, L. , Steidler, L. , Vandekerckhove, J. , and Remaut, E. (2000) Secretion of biologically active murine interleukin‐10 by *Lactococcus lactis* . Enzyme Microb Technol 27: 761–765. 11118583 10.1016/s0141-0229(00)00297-0

[mbt212781-bib-0044] Sebastian, S. , Hernández, V. , Myrelid, P. , Kariv, R. , Tsianos, E. , Toruner, M. , *et al* (2014) Colorectal cancer in inflammatory bowel disease: results of the 3rd ECCO pathogenesis scientific workshop (I). J Crohn Colitis 8: 5–18. 10.1016/j.crohns.2013.04.008 23664897

[mbt212781-bib-0045] Seet, B.T. , and McFadden, G. (2002) Viral chemokine‐binding proteins. J Leukoc Biol 72: 24–34. 12101259

[mbt212781-bib-0046] Steen, A. , Buist, G. , Leenhouts, K.J. , El Khattabi, M. , Grijpstra, F. , Zomer, A.L. , *et al* (2003) Cell wall attachment of a widely distributed peptidoglycan binding domain is hindered by cell wall constituents. J Biol Chem 278: 23874–23881. 12684515 10.1074/jbc.M211055200

[mbt212781-bib-0047] Steen, A. , Larsen, O. , Thiele, S. , and Rosenkilde, M.M. (2014) Biased and G protein‐independent signaling of chemokine receptors. Front Immunol 5: 277. 25002861 10.3389/fimmu.2014.00277 PMC4066200

[mbt212781-bib-0048] Triantafillidis, J.K. , Nasioulas, G. , and Kosmidis, P.A. (2009) Colorectal cancer and inflammatory bowel disease: epidemiology, risk factors, mechanisms of carcinogenesis and prevention strategies. Anticancer Res 29: 2727–2737. 19596953

[mbt212781-bib-0049] Triantafillidis, J.K. , Merikas, E. , and Georgopoulos, F. (2011) Current and emerging drugs for the treatment of inflammatory bowel disease. Drug Des Devel Ther 5: 185–210. 10.2147/DDDT.S11290 PMC3084301 21552489

[mbt212781-bib-0050] Trinh, R. , Gurbaxani, B. , Morrison, S.L. , and Seyfzadeh, M. (2004) Optimization of codon pair use within the (GGGGS)3 linker sequence results in enhanced protein expression. Mol Immunol 40: 717–722. 14644097 10.1016/j.molimm.2003.08.006

[mbt212781-bib-0051] Vandenbroucke, K. , de Haard, H. , Beirnaert, E. , Dreier, T. , Lauwereys, M. , Huyck, L. , *et al* (2010) Orally administered *L. lactis* secreting an anti‐TNF Nanobody demonstrate efficacy in chronic colitis. Mucosal Immunol 3: 49–56. 19794409 10.1038/mi.2009.116

[mbt212781-bib-0052] Wang, D. , Dubois, R.N. , and Richmond, A. (2009) The role of chemokines in intestinal inflammation and cancer. Curr Opin Pharmacol 9: 688–696. 19734090 10.1016/j.coph.2009.08.003 PMC2787713

[mbt212781-bib-0053] Zadravec, P. , Mavric, A. , Bogovic Matijasic, B. , Strukelj, B. , and Berlec, A. (2014) Engineering BmpA as a carrier for surface display of IgG‐binding domain on *Lactococcus lactis* . Protein Eng Des Sel 27: 21–27. 24336343 10.1093/protein/gzt059

[mbt212781-bib-0054] Zadravec, P. , Strukelj, B. , and Berlec, A. (2015a) Heterologous surface display on lactic acid bacteria: non‐GMO alternative? Bioengineered 6: 179–183. 25880164 10.1080/21655979.2015.1040956 PMC4601214

[mbt212781-bib-0055] Zadravec, P. , Strukelj, B. , and Berlec, A. (2015b) Improvement of LysM‐mediated surface display of designed ankyrin repeat proteins (DARPins) in recombinant and nonrecombinant strains of *Lactococcus lactis* and *Lactobacillus species* . Appl Environ Microbiol 81: 2098–2106. 25576617 10.1128/AEM.03694-14 PMC4345362

[mbt212781-bib-0056] Zadravec, P. , Mareckova, L. , Petrokova, H. , Hodnik, V. , Perisic Nanut, M. , Anderluh, G. , *et al* (2016) Development of recombinant *Lactococcus lactis* displaying albumin‐binding domain variants against Shiga toxin 1 B subunit. PLoS ONE 11: e0162625. 27606705 10.1371/journal.pone.0162625 PMC5015993

[mbt212781-bib-0057] Zlotnik, A. , and Yoshie, O. (2000) Chemokines: a new classification system and their role in immunity. Immunity 12: 121–127. 10714678 10.1016/s1074-7613(00)80165-x

