# Supplementary material for: Evasin‐displaying lactic acid bacteria bind different chemokines and neutralize CXCL8 production in Caco‐2 cells
Source: Microb Biotechnol. 2017 Jul 24;10(6):1732–43. doi: 10.1111/1751-7915.12781 (PMC5658612; doi:10.1111/1751-7915.12781)
Supplement: Supplementary file 1 — Table S1. Strains, plasmids and primers used in the study. Fig. S1. Shift in fluorescence intensity. Fig. S2. ELISA‐determined percentage of CCL3, CCL5 and CXCL8 removed after incubation with 6 × 10^8^ (white bars), 3 × 10^9^ (gray bars), or 6 × 10^9^ (black bars) cells/mL of recombinant L. lactis NZ9000 cells that displayed evasin‐1 (pSDEva1), evasin‐4 (pSDEva4), or evasin‐3 (pSDEva3). Fig. S3. The portion of CCL3 removed from the solution after incubation with 2 x 10^9^ Lb. salivarius cells coated with evasin‐1 (white bars) or evasin‐4 (black bars) fusion proteins. Fig. S4. ELISA‐determined CXCL8 secretion by Caco‐2 cells exposed to IL‐1β. Fig. S5. Percentage of viable Caco‐2 cells determined with trypan blue exclusion staining after 2 h of incubation with different number of L. lactis NZ9000ΔhtrA cells (2 × 10^8^, 1 × 10^9^ and 2 × 10^9^; A); or after 7 h of incubation of Caco‐2 cells with bacterial cells (L. lactis NZ9000ΔhtrA and Lb. salivarius coated with conditioned medium of L. lactis NZ9000ΔhtrA; B). Data S1. Supplemental methods. [file MBT2-10-1732-s001.docx]

# Supplemental material

**Evasin-displaying lactic acid bacteria bind different chemokines and neutralize CXCL8 production in Caco-2 cells**

Katja Škrlec^a,b^, Anja Pucer Janež^a^, Boris Rogelj^a,c,d^, Borut Štrukelj^a,e^, Aleš Berlec^a,#^

^a^ *Department of Biotechnology, Jožef Stefan Institute, Jamova 39, SI-1000, Ljubljana, Slovenia*

^b^ *Graduate School of Biomedicine, Faculty of Medicine, University of Ljubljana, SI-1000, Ljubljana, Slovenia*

^c^ *Biomedical Research Institute (BRIS), Puhova 10, SI-1000 Ljubljana, Slovenia*

^d^ *Faculty of Chemistry and Chemical Technology, University of Ljubljana, Večna pot 113, SI-1000 Ljubljana, Slovenia*

^e^ *Faculty of Pharmacy, University of Ljubljana, Aškerčeva 7, SI-1000, Ljubljana, Slovenia*

Running title:

Evasin-displaying lactic acid bacteria bind chemokines

^#^Corresponding author

E-mail: ales.berlec@ijs.si

Phone: 00 386 1 477 3754

**Supplemental tables:**

**Table S1:** Strains, plasmids and primers used in the study.

| Strain, plasmid, primer or gene | Relevant features or sequence | Reference |
| --- | --- | --- |
| Strain |  |  |
| *E. coli* |  |  |
| DH5α | endA1 glnV44 thi-1 recA1 relA1 gyrA96 deoR F^-^ Φ80d*lacZ*ΔM15 Δ(*lacZYA-argF*)U169, hsdR17(r_K_^-^ m_K_^+^), λ– | Invitrogen |
| *L. lactis* |  |  |
| NZ9000 | MG1363 *nisRK* Δ*pepN* | NIZO |
| NZ9000Δ*htrA* | NZ9000 carrying *htrA* disruption | [[1](#_ENREF_1)] |
| *Lb. salivarius* |  |  |
| ATCC 11741 | Wild type | ATCC |
| Plasmid |  |  |
| pGEM-T Easy | Ap^r^, cloning vector for PCR products | Promega |
| pGEM::Usp | pGEM-T Easy containing Usp45 signal peptide sequence (*sp*_Usp_) | [[2](#_ENREF_2)] |
| pSDLBA3b | pNZ8148 containing gene fusion of *sp*_Usp45-LEIS,_ *b-dom* and *acmA3b* | [[3](#_ENREF_3)] |
| pSDBA3b | pNZ8148 containing gene fusion of *sp*_Usp45,_ *b-dom* and *acmA3b* | This work |
| pGH::Eva1Eva3 | pGH containing *evasin-1* and *evasin-3* gene | This work |
| pGH::Eva4 | pGH containing *evasin-4* gene | This work |
| pGEM::Eva1_BamHI | pGEM-T Easy containing evasin-1 with BamHI restriction sites | This work |
| pGEM::Eva3_BamHI | pGEM-T Easy containing evasin-3 with BamHI restriction sites | This work |
| pGEM::Eva4_BamHI | pGEM-T Easy containing evasin-4 with BamHI restriction sites | This work |
| pGEM::Eva1_EcoRI | pGEM-T Easy containing evasin-1 with BamHI and EcoRI restriction site | This work |
| pGEM::Eva3_EcoRI | pGEM-T Easy containing evasin-3 with BamHI and EcoRI restriction site | This work |
| pGEM::Eva4_EcoRI | pGEM-T Easy containing evasin-4 with BamHI and EcoRI restriction site | This work |
| pNZ8148 | pSH71 derivative, P*_nisA_,* Cm^r^, nisin-controlled expression | [[4-7](#_ENREF_4)] |
| pSDEva1_B | pNZ8148 containing gene fusion of *sp*_Usp45_, e*vasin-1*, *b-dom* and *acmA3b* | This work |
| pSDEva3_B | pNZ8148 containing gene fusion of *sp*_Usp45_, e*vasin-3*, *b-dom* and *acmA3b* | This work |
| pSDEva4_B | pNZ8148 containing gene fusion of *sp*_Usp45_, e*vasin-4*, *b-dom* and *acmA3b* | This work |
| pSDEva1 | pNZ8148 containing gene fusion of *sp*_Usp45_, ev*asin-1* and *acmA3b* | This work |
| pSDEva3 | pNZ8148 containing gene fusion of *sp*_Usp45_, *evasin-3* and *acmA3b* | This work |
| pSDEva4 | pNZ8148 containing gene fusion of *sp*_Usp45_, e*vasin-4* and *acmA3b* | This work |
|  |  |  |
| Primer |  |  |
| Usp1-NcoI^a^ | 5’-ATAACCATGGCTAAAAAAAAGATTATCTCAGCTATTTTAATG-3’ | [[3](#_ENREF_3)] |
| LeisR-BamHI | 5’AGGATCCAGCATCACAGTATGATGAGATTTCAAGAGCGTAAACACCTGACAACGG-3’ | [[3](#_ENREF_3)] |
| Eva1_BamHI_F | 5'- GGATCCGAAGATGATGAAGATTATGGAGAC-3' | This work |
| Eva1_EcoRI_R | 5'- GAATTCGTTTTTTTTATCACGCCAATTTCG-3' | This work |
| Eva3_BamHI_F | 5'- GGATCCTTGGTGTCAACTATCGAAAGTCGC-3' | This work |
| Eva3_EcoRI_R | 5'- GAATTCTCGTCTTACGACGGGAGGTTCACC-3' | This work |
| Eva4_BamHI_F | 5'- GGATCCGAAGTTCCTCAAATGACATCGAG-3' | This work |
| Eva4_BamHI_R | 5'- GGATCCCCAACATTGAGCATGACGAGGTGG-3' | This work |
| Eva4_EcoRI_R | 5'- GAATTCCCAACATTGAGCATGACGAGG-3' | This work |
|  |  |  |
| Gene |  |  |
| *evasin-1* | GAAGATGATGAAGATTATGGAGACCTTGGAGGATGTCCTTTTTTAGTTGCCGAAAATAAAACAGGGTATCCAACAATCGTTGCTTGTAAACAGGATTGCAATGGAACTACAGAAACTGCTCCTAATGGAACGAGATGTTTCAGTATTGGAGACGAAGGTTTAAGGCGTATGACTGCTAATTTACCTTACGATTGCCCACTTGGCCAGTGTTCAAATGGAGATTGCATACCAAAAGAGACATATGAAGTTTGTTATCGTCGAAATTGGCGTGATAAAAAAAACGGGGGAGGTGGATCTGGAGGTGGGGGTTCTGGAGGTGGAGGAAGT | This work |
| *evasin-3* | TTGGTGTCAACTATCGAAAGTCGCACAAGTGGGGATGGGGCAGATAACTTTGACGTTGTAAGCTGTAATAAAAATTGTACGTCTGGACAGAATGAATGCCCAGAAGGATGTTTTTGCGGATTACTTGGTCAAAATAAAAAAGGTCACTGTTATAAAATTATTGGTAATCTTAGTGGTGAACCTCCCGTCGTAAGACGAGGGGGAGGTGGATCTGGAGGTGGGGGTTCTGGAGGTGGAGGAAGT | This work |
| *evasin-4* | GAAGTTCCTCAAATGACATCGAGTTCGGCTCCAGATCTTGAGGAAGAAGATGATTATACTGCATATGCTCCTTTGACTTGCTATTTCACTAATTCAACCCTTGGTCTTTTGGCTCCACCTAACTGTTCTGTATTATGTAATAGCACAACTACATGGTTTAATGAAACATCACCTAATAATGCCAGCTGTTTGTTGACAGTAGACTTTTTAACACAAGATGCAATCCTCCAAGAGAACCAACCATATAACTGTAGTGTTGGTCATTGTGATAATGGTACTTGTGCTGGACCACCTCGTCATGCTCAATGTTGG | This work |

^a^USP45 signal peptide for secretion to the growth medium contains additional alanine due to cloning reasons.

**Supplemental figures**


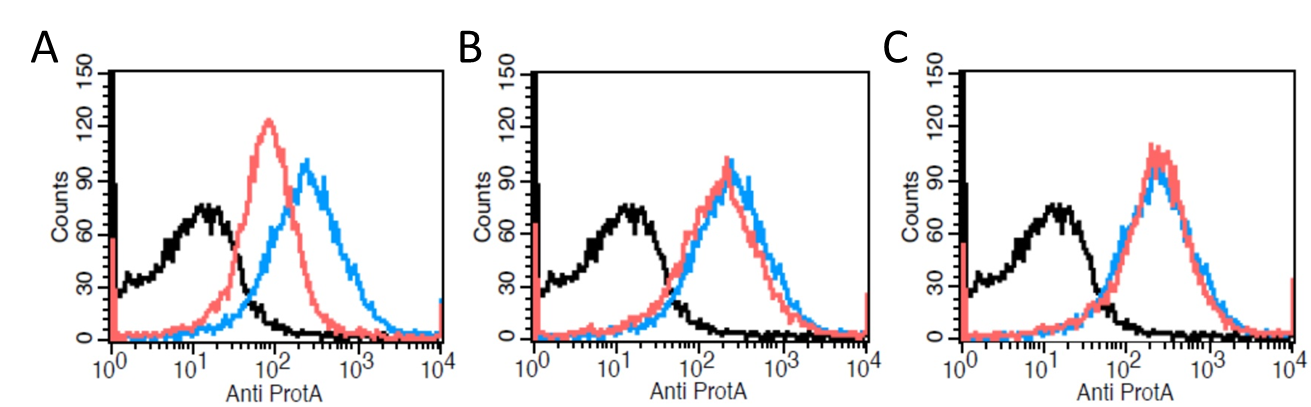


**Fig. S1:** Shift in fluorescence intensity. Black: control (pNZ8148), blue: B domain (pSDB3b), red: evasin-1_B domain (A), evasin-3_B domain (B), evasin-4_B domain (C) – expressed in *L. lactis* NZ9000*ΔhtrA* cells. Fusion proteins were detected with goat anti protein A antibody and Alexa Fluor 488-conjugated donkey anti-goat antibody.

**Fig. S2:** ELISA-determined percentage of CCL3, CCL5 and CXCL8 removed after incubation with 6 × 10^8^ (white bars), 3 × 10^9^ (gray bars), or 6 × 10^9^ (black bars) cells/mL of recombinant *L. lactis* NZ9000 cells that displayed evasin-1 (pSDEva1), evasin-4 (pSDEva4), or evasin-3 (pSDEva3). The results were from three independent experiments performed in triplicate and are expressed as mean ± SD. Significant differences (*: p<0.05; **: p<0.01; ***: p<0.001) are marked with asterisks.

**Fig. S3:** The portion of CCL3 removed from the solution after incubation with 2 x 10^9^ *Lb. salivarius* cells coated with evasin-1 (white bars) or evasin-4 (black bars) fusion proteins. Different volumes (20 mL, 10 mL, 5 mL) of *L. lactis* NZ9000*ΔhtrA* growth medium were used as a source of fusion protein. The extent of binding was normalized relative to control *Lb. salivarius* cells coated with equal volume of growth medium of *L. lactis* NZ9000*ΔhtrA* harboring empty plasmid pNZ8148. Student’s t-test was used to compare binding achieved with the same fusion proteins. Significant difference (*: p<0.05) is marked with an asterisk.


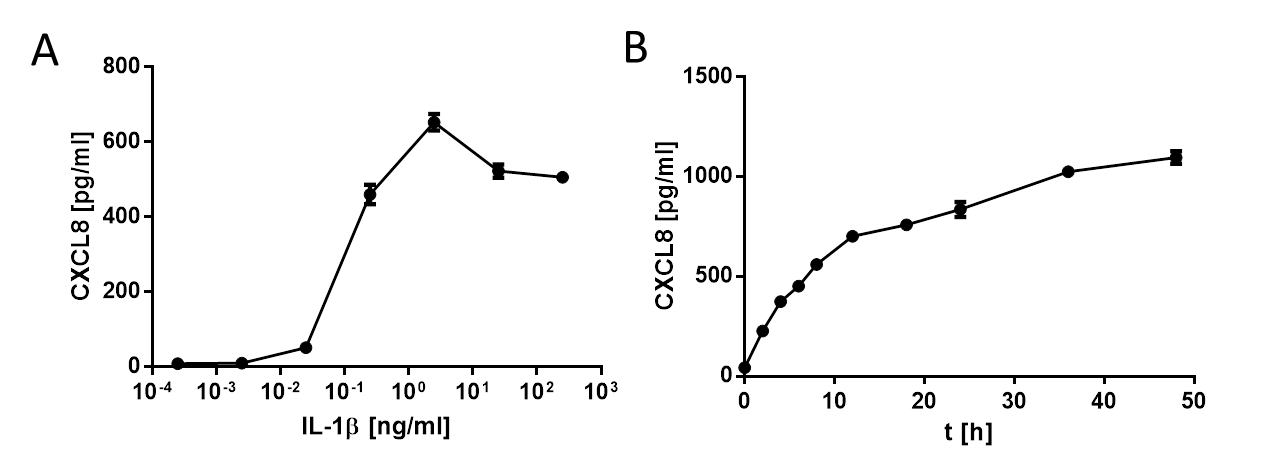


**Fig. S4:** ELISA-determined CXCL8 secretion by Caco-2 cells exposed to IL-1β. (A) CXCL8 secretion after 6 h exposure of Caco-2 cells to different concentrations of IL-1β. (B) Temporal profile of CXCL8 secretion by Caco-2 cells after stimulation with 25 ng/ml of IL-1β.


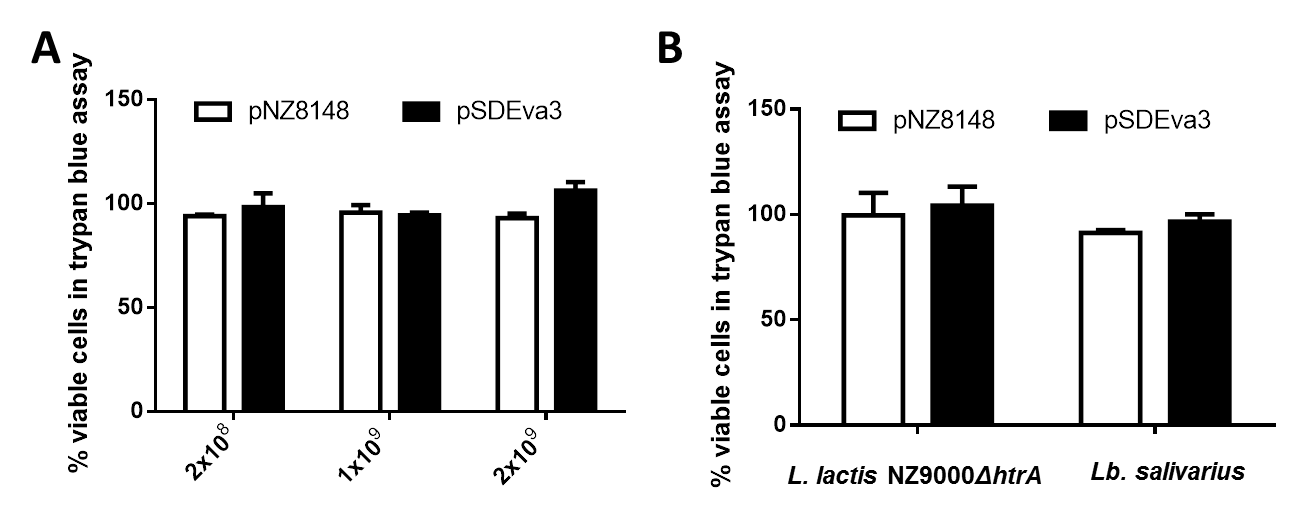


**Fig. S5:** Percentage of viable Caco-2 cells determined with trypan blue exclusion staining after 2 h of incubation with different number of *L. lactis* NZ9000*ΔhtrA* cells (2 × 10^8^, 1 × 10^9^ and 2 × 10^9^; A); or after 7 h of incubation of Caco-2 cells with bacterial cells (*L. lactis* NZ9000*ΔhtrA* and *Lb. salivarius* coated with conditioned medium of *L. lactis* NZ9000*ΔhtrA*; B).

**Supplemental methods:**

DNA manipulation and plasmid construction

Restriction enzymes and T4 DNA ligase were from Fermentas or New England Biolabs. PCR amplifications were performed with Taq polymerase (Fermentas) and KOD Hot Start Polymerase (Novagen) according to the manufacturer´s protocols. PCR products were routinely ligated to pGEM-T Easy (Promega) for sequencing and further cloning. Plasmid DNA was isolated with NucleoSpin Plasmid (Macherey-Nagel), with an additional lysozyme treatment step in the case of *L. lactis*. Electroporation of *L. lactis* was performed according to [[8](#_ENREF_8)], using Gene Pulser II apparatus (Biorad). Nucleotide sequencing was performed by GATC (Constance). Primers (IDT) and plasmids are listed in Table S1.

The *Sp_Usp45_* gene was amplified by PCR from pGEM::Usp using Usp1-NcoI/LeisR-BamHI primer pair and cloned to pSDLBA3b [[3](#_ENREF_3)] via NcoI/BamHI restriction sites, thereby removing LEISSYCDA propeptide, enabling complete processing of secretion signal, and yielding pSDBA3b. Evasin genes *evasin-1, evasin-3* and *evasin-4*, followed downstream by peptide linker (GGGGSGGGGSGGGGS) gene, were back-translated from amino acid sequences [[9](#_ENREF_9), [10](#_ENREF_10)] and codon-optimized by Gene Designer (DNA2.0) using *L. lactis* codon usage table. The genes were synthesized by ATG:biosynthetics and cloned into the plasmid for surface-display (pSDBA3b). Evasin genes flanked by BamHI sites were digested with BamHI and cloned to similarly prepared pSDBA3b*,* yielding evasin_B domain constructs pSDEva1_B, pSDEva3_B and pSDEva4_B. Primer pairs Eva1_BamHI_F/Eva1_EcoRI_R, Eva3_BamHI_F/Eva3_EcoRI_R and Eva4_BamHI_F/Eva4_EcoRI_R were used to obtain *evasin-1,* *evasin-3* and *evasin-4* genes flanked by BamHI and EcoRI sites. PCR amplicons flanked by BamHI and EcoRI sites were digested with BamHI and EcoRI and cloned to equally prepared pSDBA3b, thereby replacing the B domain gene and yielding evasin constructs pSDEva1, pSDEva3 and pSDEva4, respectively (Figure 1).

SDS PAGE and Western blot

SDS PAGE was performed with a Mini-Protean II apparatus (Bio-Rad, Hercules, USA). Samples were thawed in an ice bath, briefly sonicated with UPS200S sonicator (Hielscher), mixed with 2x Laemmli Sample buffer and dithiothreitol, and denatured by heating at 100°C before loading. Page Ruler Plus pre-stained standard (Fermentas) was used for molecular weight comparison. Proteins were transferred to nitrocellulose membrane (GE Healthcare) using wet transfer at 100 V for 90 minutes. Membranes were blocked in 5 % non-fat dried milk in TBS with 0.05% Tween-20 (TBST; 50 mM Tris-HCl, 150 mM NaCl, 0.05 % Tween 20, pH 7.5) and incubated overnight at 4°C with goat anti-protein A antibody (1:2000, Abcam) in 5 % non-fat dried milk in TBST. Following three washes with TBST, membranes were incubated for 2 h with peroxidase conjugated secondary donkey anti-goat IgG (1:5000, Jackson ImmunoResearch) in 5 % non-fat dried milk in TBST. After three further washes with TBST, membranes were incubated with Lumi-Light chemiluminescent reagent (Roche). Images were acquired using a ChemiDoc MP Imaging System (BioRad).

Flow cytometry

For flow cytometry 10 µl of a cell culture in the stationary phase was added to 500 µl of Tris-buffered saline (TBS; 50 mM Tris-HCl, 150 mM NaCl, pH 7.5) and centrifuged for 5 min at 5000 x g and 4°C. The pellet was resuspended in 500 µl of TBS and 0.2 μL of goat anti - protein A antibody (1:2500, Abcam) added. After 2 h of incubation at RT with constant shaking at 100 rpm, cells were washed three times with 200 µl 0.1 % TBS-Tween (TBST) and resuspended in 500 μL of TBS with donkey anti goat Alexa 488 antibody (Abcam, diluted 1:2500). After 2 h incubation at RT, cells were washed three times with 200 μL TBST and finally resuspended in 500 µl TBS. Samples were analysed with a FACSCalibur flow cytometer (Becton Dickinson) using excitation at 488 nm and emission at 530 nm in the FL1 channel. The results are presented as mean fluorescence intensity (MFI) values of at least 20000 lactococcal cells. The result was expressed as the average of at least three independent experiments.

Whole cell enzyme-linked immunosorbent assay (ELISA)

The whole-cell enzyme-linked immunosorbent assay (ELISA) was performed as described [1, 11]. For testing evasin surface display, 750 μL of evasin-B domain fusion-expressing *L. lactis* cell suspension in PBS with optical density A_600_ =1.0 was centrifuged (5000 x g, 5 min, 4°C) and washed twice with 500 μL PBS. Cells were then resuspended in 200 μL of goat anti-protein A antibody (Abcam, diluted 1:500 in PBS) and incubated 1 h at room temperature (RT) with gentle shaking. Cells were then washed twice with PBS and resuspended in 200 μL of peroxidase-conjugated donkey anti-goat IgG (Jackson ImmunoResearch, diluted 1:2500 in PBS). After 1 h of incubation at RT with gentle shaking, cells were washed, first with PBS and then with substrate buffer (150 mM Na_2_HPO_4_, 50 mM citric acid, pH 6.0). Cells were then resuspended in 1 mL of substrate buffer, and 100 μL of appropriate dilutions in substrate buffer (1:5 and 1:25) were loaded on a microtiter plate. 100 μL of 3,3’,5,5’-tetramethylbenzidine (TMB) substrate (Sigma-Aldrich) was added and the reaction stopped after 15 min by the addition of 50 μL of 2 M sulphuric acid. Absorbances were read at 450 nm using an Infinite M1000 (Tecan).

## ELISA for chemokine concentration determination

Standard curve ranges were 4-400 pg/mL for CXCL8, 2-200 pg/mL for CCL4, 7.81 – 500 pg/mL for CCL3, 15.6 -1000 pg/mL for CCL5, 8-1000 pg/mL for CXCL2, 12-3000 pg/mL for CXCL16, 4-1000 pg/mL for murine CXCL1 and 16-1000 pg/mL for murine CXCL2.

Nunc Maxisorp 96-well plates were coated with the recommended concentrations of chemokine binding antibodies overnight at 4°C. 100 μL samples were then added and incubated for 2 h at RT. Wells were washed five times with 200 μL of PBS containing 0.05% Tween-20 (wash buffer). 100 μL of biotinylated monoclonal antibodies against chemokine in recommended concentration were added and incubated at RT for 1 h. Wells were washed again five times with 200 μL of wash buffer. 100 μL of streptavidin-HRP or avidin-HRP diluted 1:1000 or 1:2000 was added to the plate and incubated for 1 h or 30 min at RT. The plate was washed again five times with wash buffer and 100 μL of 3,3’,5,5’-tetramethylbenzidine (TMB) substrate or 2,2'-azino-bis(3-ethylbenzothiazoline-6-sulphonic acid) (ABTS) substrate (Sigma Aldrich) was added. The TMB substrate reaction was stopped after 15 min by the addition of 50 μL of 2 M sulphuric acid. Absorbances were read at 450 nm using an Infinite M1000 microplate reader (Tecan) using wavelength correction at 650 nm.

## Luminex multiplexing system assays for chemokine concentration determination

Standard curve ranges were 119-9600 pg/mL for CXCL1, 364-88400 pg/mL for CXCL4, 59.4- 14433 pg/mL for CXCL5, 29.8-7230 pg/mL for CXCL6, 7.7-7310 pg/mL for CXCL8, 11-2680 pg/mL for CXCL16, 181-14700 pg/mL for CCL3, 121- 29310 pg/mL for CCL11, 12.7- 3080 pg/mL for CCL18, 10.8- 2630 pg/mL for CCL24 and 50.7- 12320 pg/mL for CCL25.

# Supplemental references

1. Lindholm A, Smeds A, Palva A: **Receptor binding domain of Escherichia coli F18 fimbrial adhesin FedF can be both efficiently secreted and surface displayed in a functional form in Lactococcus lactis**. *Appl Environ Microb* 2004, **70**(4):2061-2071.

2. Berlec A, Jevnikar Z, Majhenic AC, Rogelj I, Strukelj B: **Expression of the sweet-tasting plant protein brazzein in Escherichia coli and Lactococcus lactis: a path toward sweet lactic acid bacteria**. *Applied microbiology and biotechnology* 2006, **73**(1):158-165.

3. Ravnikar M, Strukelj B, Obermajer N, Lunder M, Berlec A: **Engineered lactic acid bacterium Lactococcus lactis capable of binding antibodies and tumor necrosis factor alpha**. *Appl Environ Microb* 2010, **76**(20):6928-6932.

4. de Ruyter PG, Kuipers OP, de Vos WM: **Controlled gene expression systems for Lactococcus lactis with the food-grade inducer nisin**. *Appl Environ Microbiol* 1996, **62**(10):3662-3667.

5. Kuipers OP, Beerthuyzen MM, Siezen RJ, De Vos WM: **Characterization of the nisin gene cluster nisABTCIPR of Lactococcus lactis. Requirement of expression of the nisA and nisI genes for development of immunity**. *Eur J Biochem* 1993, **216**(1):281-291.

6. Kuipers OP, de Ruyter PGGA, Kleerebezem M, de Vos WM: **Quorum sensing-controlled gene expression in lactic acid bacteria**. *J Biotechnol* 1998, **64**(1):15-21.

7. Mierau I, Kleerebezem M: **10 years of the nisin-controlled gene expression system (NICE) in Lactococcus lactis**. *Applied microbiology and biotechnology* 2005, **68**(6):705-717.

8. Holo H, Nes IF: **Transformation of *Lactococcus* by electroporation**. In: *Electroporation protocols for microorganisms.* Edited by Nickoloff JA, vol. 47. Totowa, New Jersey: Humana Press; 1995: 195-199.

9. Frauenschuh A, Power CA, Deruaz M, Ferreira BR, Silva JS, Teixeira MM, Dias JM, Martin T, Wells TN, Proudfoot AE: **Molecular cloning and characterization of a highly selective chemokine-binding protein from the tick Rhipicephalus sanguineus**. *The Journal of biological chemistry* 2007, **282**(37):27250-27258.

10. Deruaz M, Frauenschuh A, Alessandri AL, Dias JM, Coelho FM, Russo RC, Ferreira BR, Graham GJ, Shaw JP, Wells TN *et al*: **Ticks produce highly selective chemokine binding proteins with antiinflammatory activity**. *The Journal of experimental medicine* 2008, **205**(9):2019-2031.

11. Zadravec P, Strukelj B, Berlec A: **Improvement of LysM-mediated surface display of designed ankyrin repeat proteins (DARPins) in recombinant and nonrecombinant strains of Lactococcus lactis and Lactobacillus Species**. *Appl Environ Microb* 2015, **81**(6):2098-2106.
